# Medically Managed Type A Thoracic Intramural Hematoma and Penetrating Aortic Ulcer

**DOI:** 10.7759/cureus.27776

**Published:** 2022-08-08

**Authors:** Paxton P Aung, Brianna Thiessen, David Levy

**Affiliations:** 1 Emergency Medicine, Good Samaritan Hospital, New York, USA; 2 Emergency Medicine, Brookhaven Memorial Hospital Medical Center, Patchogue, USA

**Keywords:** aortic dissection, type a intramural hematoma, acute aortic syndromes, penetrating aortic ulcer, aortic intramural hematoma

## Abstract

Intramural hematoma (IMH) and a penetrating aortic ulcer (PAU) are included in a larger category of disorders termed acute aortic syndromes. These disorders typically involve the thoracic aorta, abdominal aorta, or both, and often require emergent evaluation and treatment. Both IMH and PAU, much like aortic dissection, are classified using the Stanford and DeBakey systems to indicate the aortic area involved, with Stanford type A (DeBakey type I and II) necessitating surgical intervention, and Stanford type B sufficing with medical management of blood pressure. While IMH and PAU share many characteristics of aortic dissection in terms of diagnosis and initial management, there is much controversy surrounding ultimate treatment. In this report, we describe a case of a Stanford type A IMH with associated PAU that was managed medically with a good outcome.

## Introduction

The aorta is the primary artery of the body that supplies oxygenated blood to the rest of the circulatory system and originates from the heart. It is a vascular structure comprised of three layers: the intima, the media, and the adventitia (from the innermost layer to the outermost layer), akin to an elastic tube that gives off many branch points. An acute aortic syndrome is a group of life-threatening medical conditions that involve the pathology of the aorta [1]. These conditions are rare, often fatal, and require timely diagnosis and management.

While the classic diseases of an acute aortic syndrome are aortic dissection and aortic rupture, penetrating aortic ulcer (PAU) and intramural hematoma (IMH) exist on the same spectrum and require a similar type of management strategy. The pathogenesis of PAU and IMH are believed to be related closely to hypertension and atherosclerosis. Fatty plaques accumulate on blood vessel walls and cause local inflammatory reactions that can directly damage and weaken the wall of the blood vessel [2]. Pairing this process with high blood pressure causes stress on an already weakened blood vessel wall and potentiates many of the diseases that make up acute aortic syndromes.

Penetrating aortic ulcers likely result from plaques penetrating through the intimal layer of the aorta and eventually into the media resulting in hematoma formation [3]. An IMH can develop from a PAU or from pathologic bleeding of micro blood vessels that supply the aorta itself. The management and treatment of acute aortic syndromes are guided by the areas of the aorta involved. Imaging plays a crucial role in this, and traditionally the DeBakey and Stanford classification systems have been used to delineate the affected areas. 

Initial blood pressure control remains a mainstay of therapy in all types of acute aortic syndromes, and consideration for immediate surgical intervention depends on the areas of the aortic involved. Classically, acute aortic dissections involving the ascending aorta (DeBakey A, Stanford I/II) were considered for urgent or emergent surgical intervention, and dissections involving just the descending aorta (DeBakey B, Stanford III) were treated with blood pressure control alone. These guidelines have been based on studies showing similar initial outcomes in mortality [4]. On the other hand, IMH and PAU are much rarer and compose 5% to 20% of acute aortic syndromes which are believed to occur in 2.6 to 3.5 cases per 100,000 person-years [5,6]. Given the rarity of these diseases, the ideal management remains up for debate. In this article, we present a case of a patient with type A acute aortic IMH that had a good outcome with medical management alone.

## Case presentation

A 58-year-old male presented to the emergency department (ED) with several hours of left-sided chest pain, radiating to his left shoulder and left back. The pain was described as sharp, progressive in severity, and without any specific modifying factors. He denied any nausea, vomiting, cough, fever, shortness of breath, motor or sensory deficits, and sick contacts. His medical history consisted only of hypertension with medication noncompliance.

The patient’s initial vital signs in the ED were a pulse rate of 87 beats per minute, blood pressure of 220/131 mm Hg, respiratory rate of 20 breaths per minute, oxygen saturation of 93% on room air, and a temporal temperature of 96°F. The physical exam demonstrated a well-developed, overweight, uncomfortable appearing man. His skin was warm and diaphoretic but appropriate for his age and ethnicity. The rest of the exam was largely unremarkable. He had a normal heart rate, normal heart sounds, normal breath sounds with appropriate and symmetric chest excursion, and no chest wall tenderness. His abdomen was soft, non-tender, non-distended, and without any peritoneal signs. He was ambulating well, had intact sensation in all extremities, and moving all extremities equally with the full range of motion and strength.

**Figure 1:**
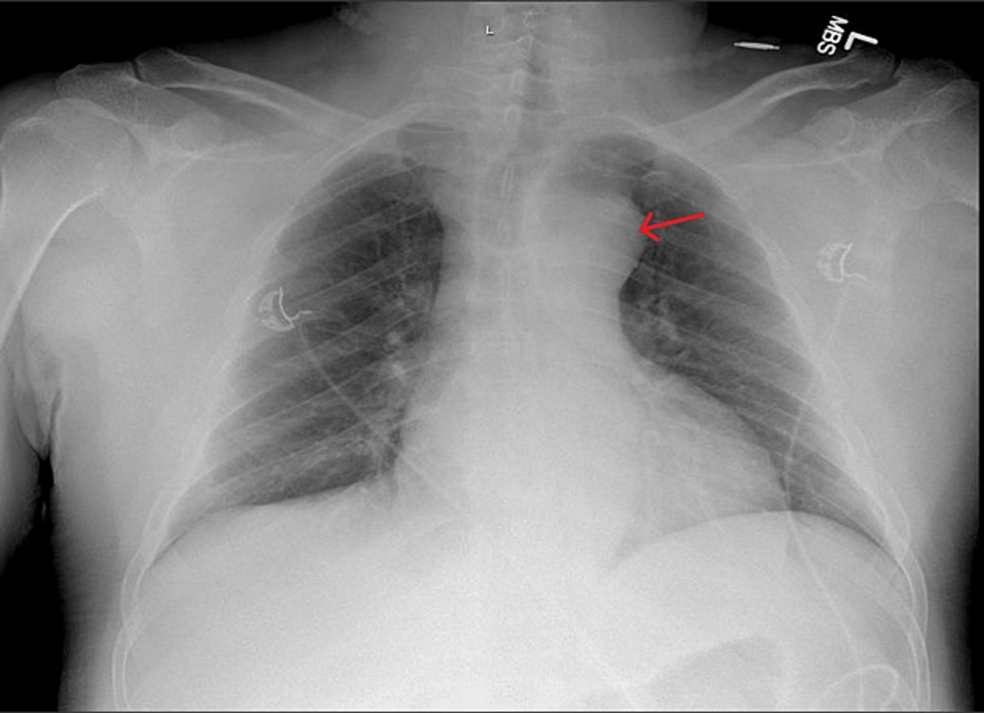
A supine anteroposterior view of the patient’s chest. The aortic knob appears enlarged (red arrow).

The laboratory studies were largely unremarkable with most electrolyte values, white blood cell count, and troponin levels within normal limits. The patient’s electrocardiogram showed a regular sinus rhythm at a rate of about 90 beats per minute with normal axis, intervals. There were no concerning ST segment or T-wave changes suggestive of acute ischemia. A portable chest X-ray was obtained as the initial imaging modality and was significant for an enlarged appearance of the cardiac and mediastinal contours (Figure 1).

**Figure 2:**
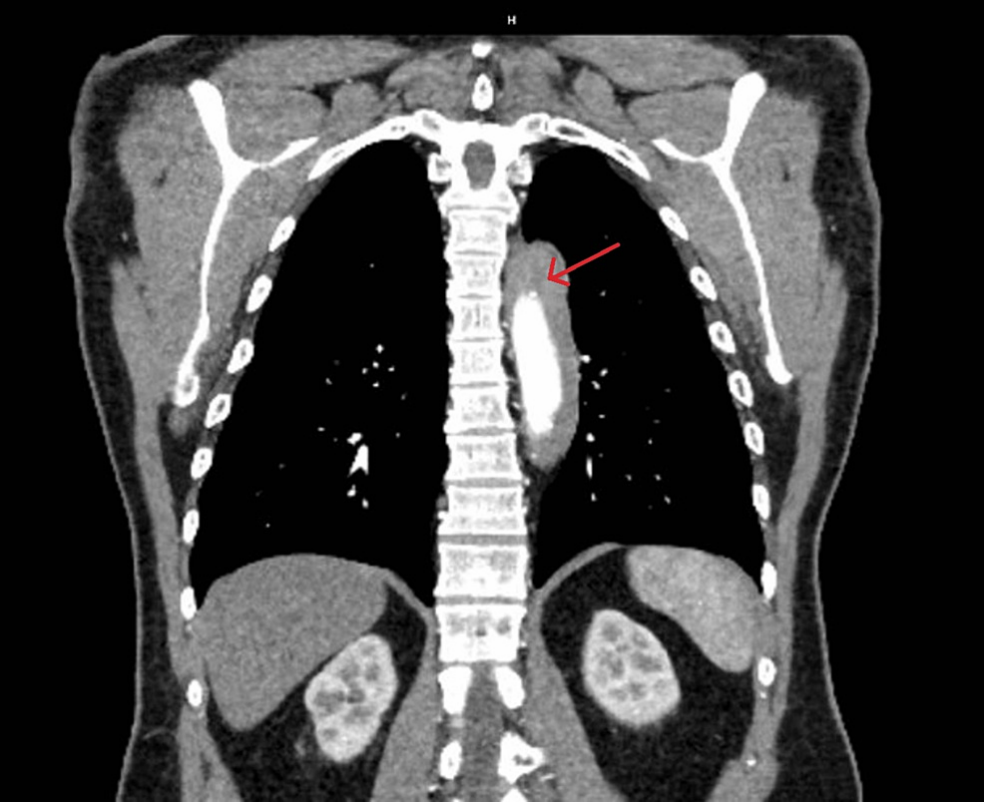
CT imaging showing a coronal view of the intramural hematoma surrounding the descending thoracic aorta (red arrow).

**Figure 3:**
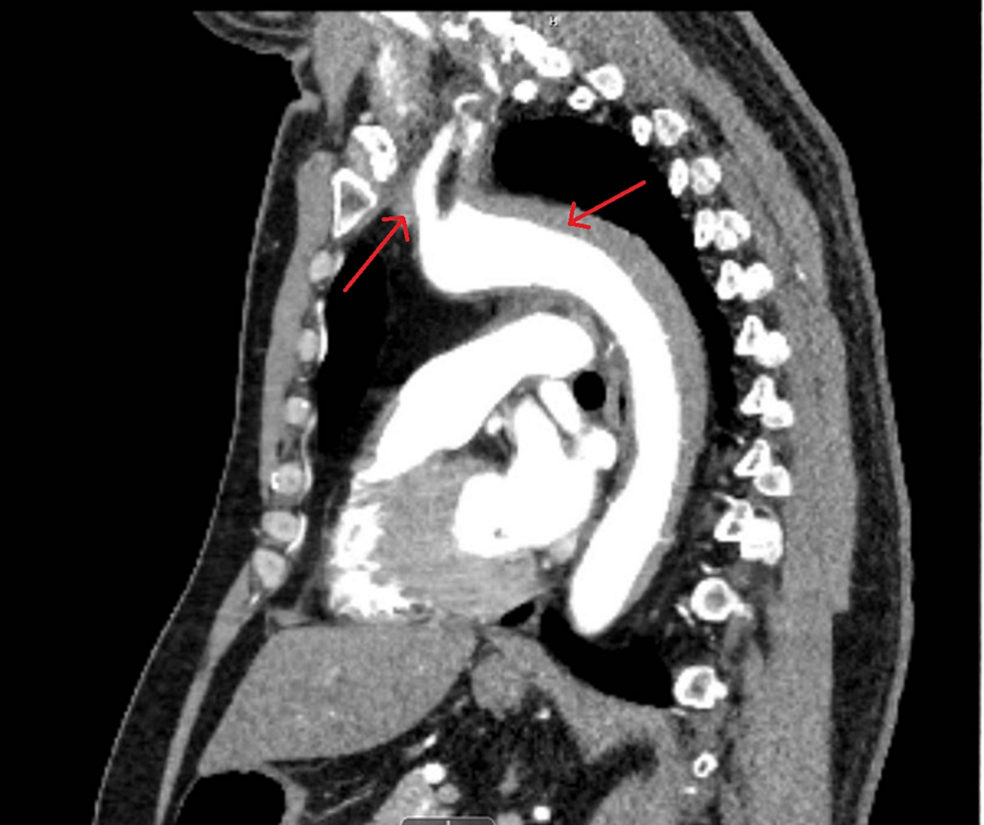
CT imaging showing a sagittal view of the intramural hematoma tracking proximally from the arch of the aorta and distally (red arrows).

**Figure 4:**
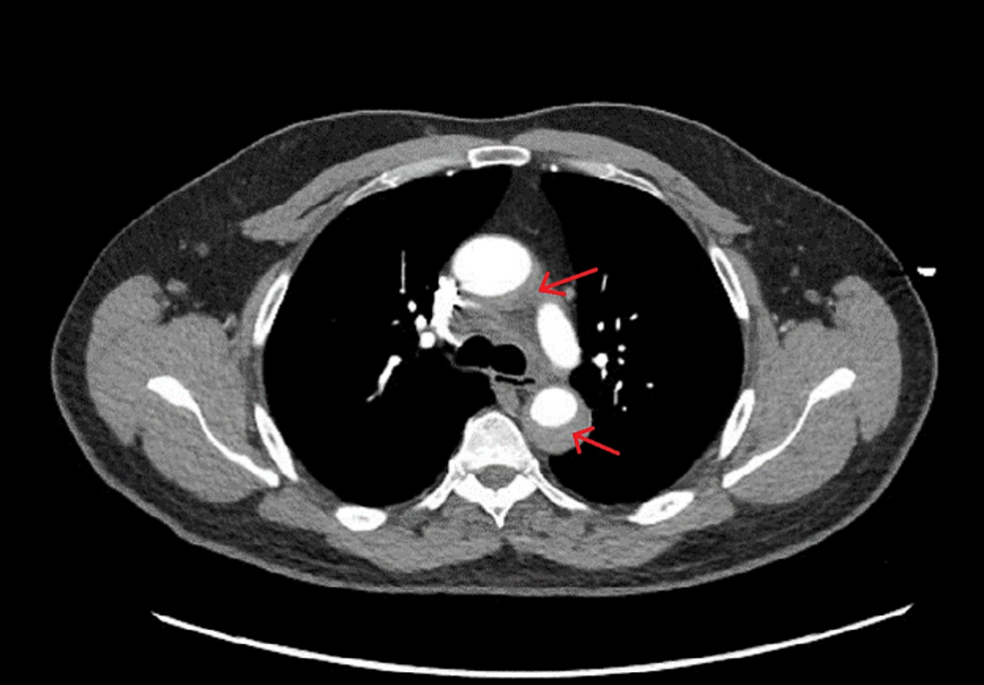
CT imaging showing an axial view of the intramural hematoma surrounding the lumen of the aorta (red arrows).

The patient was then expeditiously taken over for computed tomography (CT) angiography imaging of the chest, abdomen, and pelvis and was found to have an IMH extending from the origin of the brachiocephalic artery to the distal thoracic aorta (Figures 2, 3, 4). There was no evidence of intraluminal flaps or contrast extravasation into the hematoma that would suggest an aortic dissection or communication between the lumen of the aorta and the hematoma itself.

Concurrently, medical therapy with 20 mg intravenous pushes of labetalol, a non-selective beta blocker, was initiated in the ED to lower the patient’s blood pressure and heart rate. The labetalol was then switched to a continuous esmolol infusion. When the patient’s blood pressure was not adequately controlled on maximum doses of esmolol, a continuous infusion of clevidipine, a dihydropyridine calcium channel blocker that acts to dilate arteries, was initiated, allowing for more precise control of the patient’s hemodynamic status.

The cardiothoracic surgery team was consulted, immediate surgical intervention was deemed not necessary, and the patient was admitted to the cardiothoracic intensive care unit for blood pressure control. The patient’s blood pressure was managed with continuous labetalol and clevidipine infusions, titrated to a goal systolic blood pressure of less than 110 mm Hg. Repeat CT imaging on day two of his hospitalization showed a stable hematoma. The patient remained in the intensive care unit for four days and tolerated being transitioned to oral antihypertenbeforeior to discharge.

A repeat outpatient CT angiography imaging 11 days after admission showed that the hematoma appeared stable without any development of dissection flaps or contrast extravasation between the lumen of the aorta and the intramural hematoma, although there was a mild increase in the size of the diameter of the hematoma ranging from 2 mm to 4 mm. Cardiothoracic surgery continued to treat the patient conservatively, with blood pressure and heart rate control and close outpatient follow-up with serial imaging studies.

## Discussion

As previously mentioned, acute aortic syndromes share many similarities in their pathophysiology. Atherosclerosis and hypertension play a large role in the development of IMH, PAU, aortic dissections, and ruptures, but the exact mechanism that leads to these diseases remains uncertain. While intramural aortic hematomas share similar pathophysiology to other aortic syndromes, they are distinct entities that exist on a spectrum. An IMH can lead to many of the same complications as other aortic syndromes, with the most worrisome complication being a compromised hemodynamic status. This is largely attributed to several reasons including the extension of dissection or hematoma into the pericardium of the heart, causing cardiac tamponade, or aortic rupture leading to massive hemorrhage [7].

Although many earlier case reports of type A IMH showed high rates of mortality without operative intervention, as high as 66% in some cases [8], later case reports and retrospective studies have shown mortality to be as low as 0% to 8% in medical management alone with reabsorption of the hematoma in as many as 67% of patients, and five-year survival rates in the range of 80% to 85% [9,10]. As we learn more about these subsets of acute aortic syndromes, more contention has risen on the ideal timing of surgical intervention, and even the necessity of surgery during the same admission.

Some determining factors can help clinicians distinguish patients that will benefit from early surgery from those that can have delayed interventions. These include signs and symptoms consistent with ischemia or circulatory compromise such as ongoing pain, syncopal episodes, neurologic deficits, hypotension, and tachycardia despite the initiation of anti-hypertensives and medications to control the heart rate [7]. 

Regardless of these factors and presentation, consultation with the appropriate surgical specialists and hemodynamic management is, without a doubt, standard in the treatment of all acute aortic syndromes. However, it is also important for clinicians to consider IMH as a distinct entity from classical dissections that may benefit even further from maximal medical therapy and subsequent expectant management with delayed surgery or surgery as needed. Patient characteristics and presentation play a large role in making this determination with differing recommendations based on geographical location [11]. The patient in our case benefitted from early, aggressive blood pressure control leading to a stable hemodynamic status that precluded the need for any surgical intervention during their admission. More research is needed to determine the ideal course of treatment but for now, a case-by-case approach with adherence to maximizing medical therapy seems to work best. 

## Conclusions

Acute aortic syndromes represent life-threatening emergencies that require timely diagnosis and treatment. Aortic IMH and PAU are important differentials to consider when patients present with signs and symptoms suggestive of an aortic dissection. While very similar, there are nuances to consider that could potentially change the course of their treatment during admission. These can include the characteristics of the patient, signs, symptoms, and their severity upon presentation, along with responsiveness to medical therapy. 

As we continue to learn more about their pathophysiology and management with improving diagnostic and therapeutic modalities, the ideal strategy for timing and the need for operative intervention continues to evolve. This case report serves to highlight two key points that remain constant in treating this subset of acute aortic syndromes. One is to take a patient-centered approach, and two is to maximize medical therapy with early hemodynamic control to result in the best outcomes. 
